# Myocardial recovery in peri-partum cardiomyopathy after continuous flow left ventricular assist device

**DOI:** 10.1186/1749-8090-6-150

**Published:** 2011-11-14

**Authors:** Lars H Lund, Karl-Henrik Grinnemo, Peter Svenarud, Jan van der Linden, Maria J Eriksson

**Affiliations:** 1 Department of Cardiology, Karolinska University Hospital, Stockholm, Sweden; 2 Department of Cardiothoracic Surgery and Anaesthesiology, Karolinska University Hospital, Stockholm, Sweden; 3 Department of Clinical Physiology, Karolinska University Hospital, Stockholm, Sweden

**Keywords:** Peri-partum cardiomyopathy, heart failure, recovery, left ventricular assist device, mechanical circulatory support

## Abstract

Left ventricular assist devices (LVADs) offer effective therapy for severe heart failure (HF) as bridge to transplantation or destination therapy. Rarely, the sustained unloading provided by the LVAD has led to cardiac reverse remodelling and recovery, permitting explantation of the device. We describe the clinical course of a patient with severe peri-partum cardiomyopathy (PPCM) rescued with a continuous flow LVAD, who experienced recovery and explantation. We discuss assessment of and criteria for recovery.

## Background

### Peri-partum cardiomyopathy

Peri-partum cardiomyopathy (PPCM) affects one in 300 to one in 100,000 pregnant patients, depending on ethnic origin [1]. Risk factors include previous episode of PPCM, multiparity and African ancestry. Causes are poorly understood but prolactin and/or immune-mediated mechanisms may be important. Therapy is supportive although specific therapy with bromocriptine may be beneficial. Prognosis is variable. In those that survive without transplantation, LVEF may improve but generally does not normalize [1].

### Recovery with LVAD

In non-ischemic cardiomyopathy, myocardial injury may be reversible. Sustained LV unloading from pulsatile devices coupled with aggressive reverse-remodeling pharmacologic therapy, possibly together with the β_2_-agonist clenbuterol (the HARPS protocol), may permit reversal of the molecular, cellular and structural remodeling seen in HF, and clinical recovery [2]. However, in most reports, recovery is rare and often not sustained [3,4], and PPCM and severe mitral regurgitation have not been studied [2,5]. Recovery is thought to occur mainly with pulsatile devices, but recently the HARPS protocol with clenbuterol achieved success also with continuous flow devices [5].

### Criteria for and assessment of recovery

Recovery with device and prognosis after explant are unpredictable. Prior to implantation, younger age and shorter duration of HF but not LVEF or LVEDD predict recovery [4,6]. Assessment of recovery requires turning the LVAD "off". Our protocol for the HeartMate II entails ensuring an INR ≥ 2.0, titrating down to 8,000 rpm, administering intravenous heparin (200 units/kg) and ensuring an activated clotting time > 400 at all times that the rpm is below 8,000, followed by gradual titration down to 6,000 rpm. This pump speed approximates zero forward flow [7]. Echocardiography, invasive hemodynamics and the cardiopulmonary exercise test are performed at 6,000 rpm and LVEF > 45 and LVEDD < 55 mm coupled with preserved hemodynamics suggest recovery [4,6]. The HARPS criteria have been established as criteria for recovery (http://clinicaltrials.gov identifier NCT00585546) (table 2) . Our patient met all HARPS criteria except peak VO_2 _and ventilatory equivalent for CO_2 _(VE/VCO_2_). We considered the peak VO_2 _adequate and attributed the very high VE/VCO_2 _to anxiety. The patient met several additional criteria for recovery described by Dandel *et al. *[6].

### Recovery in PPCM

LVAD-induced recovery in PPCM has to our knowledge been described only in a handful of patients and all with older pulsatile devices [8-10], and was excluded in the series of Birks *et al. *[2,5]. Furthermore, right ventricular disease is more severe in PPCM than in idiopathic dilated cardiomyopathy [11] and pulsatile devices unload the right ventricle more effectively than do non-pulsatile devices, suggesting both that the benefits of an LVAD, especially non-pulsatile, and the potential for recovery, may be lower in PPCM. To our knowledge, recovery with a non-pulsatile device has not previously been described. Important for recovery is aggressive reverse remodelling medical therapy, and assessment of recovery requires down-titration of the pump coupled with invasive and exercise testing.

We describe the clinical course of severe PPCM rescued with a continuous flow LVAD, who experienced recovery and explantation. We discuss assessment of and criteria for recovery.

## Case Report

### Pre-LVAD

The patient is a 37-year old African-American woman, gravida 2 para 2, who presented to the Emergency Department 8 days after normal spontaneous delivery with severe dyspnea, pink frothy sputum and a respiratory rate of 44 per minute.

Blood pressure was 145/105 mm hg, heart rate regular at 105 per minute, O2 saturation was 88% on room air and the patient was afebrile. Exam revealed decreased breath sounds bilaterally and a faint systolic murmur at the apex. EKG revealed sinus tachycardia. Troponin T was < 0.01 microg/L, NT-proBNP was 2060 ng/L and D-dimer was 9.7 mg/L. Computed tomography of the chest revealed widened vessels and mild bilateral pleural effusions, but no pulmonary embolism, and a cursory echocardiogram revealed left ventricular ejection fraction (LVEF) of 10-15% and moderate mitral regurgitation. The patient was intubated and transferred to the thoracic intensive care unit (ICU).

In the ICU, hemodynamics deteriorated, systolic blood pressure was 70 mm Hg, LVEF was 5-10% and right ventricular function deteriorated, and peripheral veno-arterial extracorporeal membrane oxygenation (ECMO) was instituted emergently. ECMO could not be weaned although right ventricular function improved, and on day 4, a continuous flow long-term left ventricular assist device (LVAD, HeartMate II, Thoratec, Pleasanton, CA, USA) was implanted as a bridge to transplant. Patient data are listed in table 1.

**Table 1:** Clinical data at implantation, weaning and post-explantation

Time/Parameter	Pre ECMO	3 months post LVAD	9000 rpm rest	6000 rpm rest	9000 rpm exercise	6000 rpm exercise	p-explant	9 months p-explant
NYHA	IV	I-II	I-II	I-II	--	--	--	I-II

**Hemodynamics**								

Systolic blood pressure, mm Hg	70	--	--	--	--	--	80	90

Mean blood pressure, mm Hg	40	95	80	85	180	130	-	-

Diastolic blood pressure, mm Hg	-	--	--	--	--	--	50	60

Heart rate, beats/min	130	66	70	85	182	185	110	73

Cardiac index, L/min/m2	-	-	-	3.2	-	4.9	2.9	--

Pulmonary capillary wedge, mm Hg	-	-	-	11	-	22	-	--

Pulmonary artery systolic, mm Hg	-	-	-	21	-	40	28	--

Pulmonary artery diastolic, mm Hg	-	-	-	10	-	20	11	--

Central venous pressure, mm Hg	-	-	-	4	-	11	13	--

Right ventricular stroke work index, g/m^2^	-	-	-	366	-	416	87	-

Mixed venous O_2 _saturation, %	-	-	-	67	-	27	61	--

**LVAD monitor**								

Revolutions per minute	--	-	9000	6000	9000	6000	--	--

Flow, L/min	--	-	5.3	"---"	6.6	"---"	--	--

Pulsatility index	--	-	5.4	6.2	3.7	6.3	--	--

**Exercise test**								

VO_2_, ml/(kg × min)	--	--	--	--	--	15.3	--	15.5

VE/VCO_2_	--	--	--	--	--	75	--	35

Respiratory exchange ratio	--	--	--	--	--	1.01	--	0.9

**Laboratory**								

Creatinine, μmol/L	64	109	61	-	-	-	124	95

NT-proBNP, pg/mL	2060	-	137	-	-	-	6530	312

Troponin T, ng/mL	< 0.01	-	< 0.01	-	-	-	0.90	-

**Echocardiography**								

Left ventricular ejection fraction, %	10-15	70-75	75-80	70-75	75	70	60-70	55-60

Left ventricular end-diastolic diameter, mm	-	42	37-40	39	42	37-40	35	51

Fractional shortening, %	-	40	50	40	48	45	37	31

Right ventricular fractional area change, %	-	35	38	40	-	39	-	32

Right ventricular end-diastolic diameter, mm	-	28	27	27	23	22	27	29

Tricuspid regurgitation, grade, 1-4	-	2	< 1	< 1	-	< 1	2	2

Tricuspid annular plane systolic excursion, mm	-	8.5	9	9.5	-	10	11	15

E/A ratio	-	2.2	1.6	2.8	-	1.8	1.6	2.5

E/E' ratio	-	-	12	15	-	11	-	18

Left atrial diameter, mm	-	25	31	31	32	30	23	46

Mitral regurgitation, grade, 1-4	3	1	1	1	-	1	1	3

Septum during systole	-	right	right	left	right	left	left	left

Aortic valve opening	1/1	1/1	1/1	1/1	1/1	1/1	1/1	1/1

- not available

-- not applicable

"---" when flow is low, the HeartMate II controller does not estimate flow and the monitor instead displays "---"

### Post-LVAD

Post-operative course was uneventful. The patient was treated with aspirin 160 mg daily, warfarin adjusted to an international normalized ratio (INR) of 2-3, and ramipril, metoprolol, spironolactone and furosemide. She engaged in structured aerobic exercise training 3 times per week.

At 6 months post-implantation the patient was in NYHA I and we designed several weaning trials. We performed echocardiography, invasive hemodynamics and cardiopulmonary exercise testing with the pump set at baseline 9,000 revolutions per minute (rpm) and down-titrated to 6,000 rpm, with full heparinization (table 1). The patient met all Harefield Recovery Protocol Study (HARPS) criteria (table 2) except peak VO_2 _and ventilatory equivalent for CO_2 _(VE/VCO_2_). We considered the peak VO_2 _adequate and attributed the very high VE/VCO_2 _to anxiety.

**Table 2:** HARPS criteria.

Left ventricular end-diastolic diameter	< 6 cm
Left ventricular end-systolic diameter	< 5 cm
Left ventricular ejection fraction	> 45%
Pulmonary capillary wedge pressure	< 12 mm Hg
Cardiac Index	> 2.8 L/min/m^2^
Peak VO_2 _(exercise)	> 16 ml/kg/min
Ventilatory equivalent of CO_2 _(exercise)	< 34

All criteria should be met with pump "off" (6000 rpm for HeartMate II)

### Post LVAD explantation

Explantation was performed through median sternotomy and left-sided thoracotomy on cardiopulmonary bypass and a fibrillating heart. The inflow canula was removed, the inside of the left ventricle was inspected for thrombus, and the defect in the left ventricle was sutured directly. The outflow graft was cut and sutured near the aorta. The patient was treated with milrinone, levosimendan and inhaled nitric oxide prophylactically. Ramipril and metoprolol were restarted on day 4 and the patient was discharged on day 32.

At last follow up, 18 months post explant (table 1), she has remained stable in NYHA I-II. The degree of secondary mitral regurgitation has worsened somewhat, due to an asymmetrical LV contraction pattern, even though QRS complexes remain narrow. Future follow-up unless otherwise indicated will consist of monthly physician visits, echocardiography every 3 months and cardiopulmonary exercise testing every 6 to 12 months. The patient is aware of the risk of gradual or even acute deterioration and prepared for mitral valve intervention or heart transplantation should this become necessary.

## Conclusions

PPCM is uncommon but potentially severe. Recovery may occur spontaneously but with cardiogenic shock prognosis is poor. Recovery after LVAD placement is poorly described and PPCM has been excluded from most recovery series. Our observations raise the possibility of improving recovery and prognosis in PPCM with early implantation of LVAD, perhaps also in moderately severe cases.

## Consent

The patient has provided consent for this case report to be published.

## Competing interests

LHL and PS have received speakers and/or consulting fees and/or research grants from Thoratec and HeartWare, manufacturers of assist devices.

## Authors' contributions

Author contributions were as follows: LHL: study conception, data collection, analysis, interpretation, manuscript; KHG: data collection, analysis, manuscript revision; PS: data interpretation, manuscript revision; JVDL: data interpretation, manuscript revision; MJE: data collection, analysis, interpretation, manuscript revision. All authors have read and approved the final manuscript.
